# Digital Health Intervention to Reduce Malnutrition Among Individuals With Gastrointestinal Cancer Receiving Cytoreductive Surgery Combined With Hyperthermic Intraperitoneal Chemotherapy: Feasibility, Acceptability, and Usability Trial

**DOI:** 10.2196/67108

**Published:** 2025-04-07

**Authors:** Yu Chen Lin, Ryan Hagen, Benjamin D Powers, Sean P Dineen, Jeanine Milano, Emma Hume, Olivia Sprow, Sophia Diaz-Carraway, Jennifer B Permuth, Jeremiah Deneve, Amir Alishahi Tabriz, Kea Turner

**Affiliations:** 1 Department of Health Outcomes and Behavior, Moffitt Cancer Center, Tampa, FL, United States; 2 Department of Surgery, University of Maryland School of Medicine, Baltimore, MD, United States; 3 Department of Gastrointestinal Oncology, Moffitt Cancer Center, Tampa, FL, United States; 4 Department of Rehabilitation Services, Moffitt Cancer Center, Tampa, FL, United States; 5 Department of Cancer Epidemiology, Moffitt Cancer Center, Tampa, FL, United States; 6 Department of Surgery, University of North Carolina at Chapel Hill School of Medicine, Chapel Hill, NC, United States; 7 Division of Health Systems, Policy, and Innovations, University of North Carolina at Chapel Hill School of Nursing, Chapel Hill, NC, United States; 8 UNC Lineberger Comprehensive Cancer Center, Chapel Hill, United States

**Keywords:** gastrointestinal cancer, peritoneal disease, cytoreductive surgery combined with hyperthermic intraperitoneal chemotherapy, digital health intervention, nutrition, feasibility

## Abstract

**Background:**

Cytoreductive surgery combined with hyperthermic intraperitoneal chemotherapy (CRS-HIPEC) can improve survival outcomes for individuals with gastrointestinal (GI) cancer and peritoneal disease (PD). Individuals with GI cancer and PD receiving CRS-HIPEC are at increased risk for malnutrition. Despite the increased risk for malnutrition, there has been limited study of nutritional interventions for individuals receiving CRS-HIPEC.

**Objective:**

We aimed to test the feasibility, acceptability, and usability of Support Through Remote Observation and Nutrition Guidance (STRONG), a multilevel digital health intervention to improve nutritional management among individuals with GI cancer and PD receiving CRS-HIPEC. We also assessed patient-reported outcomes, including malnutrition risk, health-related quality of life, and weight-related measures.

**Methods:**

STRONG is a 12-week digital intervention in which participants received biweekly nutritional counseling with a dietitian, logged food intake using a Fitbit tracker, and reported nutrition-related outcomes. Dietitians received access to a web-based dashboard and remotely monitored patients’ reported food intake and nutrition-impact symptoms. Implementation outcomes were assessed against prespecified benchmarks consistent with benchmarks used in prior studies. Changes in patient-reported outcomes at baseline and follow-up were assessed using linear and ordered logistic regressions.

**Results:**

Participants (N=10) had a median age of 57.5 (IQR 54-69) years. Feasibility benchmarks were achieved for recruitment (10/17, 59% vs benchmark: 50%), study assessment completion (9/10, 90% vs benchmark: 60%), dietitian appointment attendance (7/10, 70% vs benchmark: 60%), daily food intake logging adherence (6/10, 60% vs benchmark: 60%), and participant retention (10/10, 100% vs benchmark: 60%). Most participants rated the intervention as acceptable (8/10, 80% vs benchmark: 70%) and reported a high level of usability for dietitian services (10/10, 100%). The benchmark usability for the Fitbit tracker to log food intake was not met. Compared to baseline, participants saw on average a 6.0 point reduction in malnutrition risk score (*P*=.01), a 20.5 point improvement in general health-related quality of life score (*P*=.002), and a 5.6 percentage point increase in 1-month weight change (*P*=.04) at the end of the study.

**Conclusions:**

The STRONG intervention demonstrated to be feasible, acceptable, and usable among individuals with GI cancer and PD receiving CRS-HIPEC. A fully powered randomized controlled trial is needed to test the effectiveness of STRONG for reducing malnutrition and improving patient outcomes.

## Introduction

Malnutrition is commonly observed among individuals with gastrointestinal (GI) cancer and can severely affect disease prognosis, quality of life, and survival [1,2]. Individuals with GI cancer are at high risk of developing peritoneal disease (PD), the metastasis of cancer to the abdominal cavity, which occurs in about 40% of patients with GI cancer [3]. Cytoreductive surgery combined with hyperthermic intraperitoneal chemotherapy (CRS-HIPEC) can offer survival benefits for individuals with GI cancer and PD [4,5]. CRS-HIPEC is a 2-step approach that removes all visible cancerous tumors in the abdomen through a surgical procedure, followed by heated chemotherapy during surgery [5]. Due to the invasive nature of this extensive operation, postoperative morbidities are common, including weight loss, which occurs in more than 90% of individuals receiving CRS-HIPEC [6]. Malnutrition, arising from loss of appetite and malabsorption, occurs in about 50% to 60% of individuals receiving CRS-HIPEC, which can negatively affect postoperative outcomes (eg, length of hospital stay, hospital readmission, and mortality) [7-10]. After CRS-HIPEC, patients often experience a decline in nutritional status, heightening the importance of adequate nutritional support in the postoperative period [6,11].

Medical nutrition therapy (MNT), which includes dietitian-led nutritional counseling and additional dietary interventions, has been shown to improve nutritional outcomes for individuals with GI cancer [12,13]. However, multilevel barriers hinder access to MNT and its effectiveness. At the system level, there may be limited outpatient services for nutritional counseling, fragmented oncology and nutritional care, and inconsistent nutrition-screening procedures across clinics [14,15]. For example, a survey among surgical oncologists who specialize in CRS-HIPEC showed that only one-third of providers reported the availability of malnutrition screening at their practice [16]. At the provider level, available dietitians may be lacking, and MNT is not routinely provided to individuals with cancer [14,17,18]. There are also many barriers at the patient level, including the lack of adherence to nutritional programs due to clinical factors (eg, difficulties swallowing, fatigue, nausea, and pain) and nonclinical factors (eg, lack of motivation and time constraint) [19]. Digital nutritional interventions, such as remote monitoring, can help patients overcome barriers to accessing and adhering to traditional nutritional interventions and can improve patient outcomes [20,21]. However, research on digital nutritional interventions for individuals with cancer is limited [22]. There is a need to develop and test digital nutritional interventions, particularly for individuals receiving CRS-HIPEC who are at high risk for malnutrition.

To address this gap, the goal of this study is to pilot test the Support Through Remote Observation and Nutrition Guidance (STRONG) intervention, a multilevel digital health intervention to improve nutritional outcomes. The study aims (1) to assess the feasibility, acceptability, and usability of the STRONG intervention for individuals with GI cancer and PD undergoing CRS-HIPEC and (2) to evaluate patient-reported outcomes, including malnutrition risk, health-related quality of life, and weight-related measures. To the best of our knowledge, this is the first digital nutritional intervention conducted among individuals receiving CRS-HIPEC, who are at high risk of postoperative malnutrition and face unique barriers to accessing and using MNT [7,20,21]. Findings from this study will inform broader interventions to manage cancer-related malnutrition and guide a future randomized controlled trial to evaluate the impact of the STRONG intervention.

## Methods

### Study Design

We conducted a single-arm feasibility trial of STRONG, a 12-week digital intervention to improve postoperative nutrition. Guided by the Obesity-Related Behavioral Intervention Trials model, the goal of the single-arm study was to identify potential technical issues with digital health delivery, assess the optimal length of intervention delivery, and gather participant feedback on acceptability to inform intervention refinement prior to larger testing in a randomized trial [23]. The intervention was developed based on the Theoretical Domains Framework, a theory used to understand and address multilevel behavior change (ie, patient and clinician behavior) in health care settings [24]. Participants received biweekly MNT (6 sessions) that included nutritional counseling with a registered dietitian and continuous remote monitoring of participants’ dietary needs by the dietitian. In addition, participants logged daily food intake using a Fitbit device (Inspire 2) and completed 5 study assessments related to patient malnutrition, nutrition-related symptoms, and quality-of-life outcomes (at baseline and 4, 8, 12, and 16 weeks after study enrollment). Participants provided feedback on the intervention’s acceptability and usability (at week 12).

### Participants

Individuals who met the following criteria were eligible to participate in the study: (1) older than 18 years, (2) diagnosed with primary GI cancer, (3) diagnosed with PD, (4) underwent curative-intent CRS-HIPEC at Moffitt Cancer Center (Moffitt; with cytoreduction completeness score of 0‐1), (5) transitioned to a postoperative oral diet, (6) were able to speak and read English, and (7) provided informed consent. Individuals were excluded from the study if they met any of the following criteria: (1) had documented or observable psychiatric or neurological condition that would inhibit with study participation, (2) were undergoing treatment for another primary cancer, and (3) received postoperative parenteral or enteral nutrition.

### Recruitment

Potential participants were identified through a collaboration between Moffitt’s GI clinic staff and the study coordinators. In addition, we screened the patients’ electronic health records (EHRs) to determine their eligibility. Eligible participants were contacted by phone, unencrypted email, videoconference, or in-person meetings to introduce them to the study and determine their interest in participating. The participants who provided informed consent were given study materials and equipment during patient visits or by mail, including (1) a welcome packet and checklist describing the study components, instructions on using and caring for the Fitbit tracker and tablet, and brief instructions on estimating food portion size; (2) a Fitbit tracker; and (3) a study loaned tablet to log daily food intake with the Fitbit application already downloaded and synced. Participants also had the choice of downloading the Fitbit application on a personal device if preferred. Within 3 to 5 days of the participant receiving the Fitbit tracker, one of the study coordinators (RH, OS, and SD-C) contacted the participant to confirm that they were able to use the device. Participants received an introduction to the Fitbit tracker before undergoing CRS-HIPEC. Recruitment occurred from December 2022 to July 2023.

### Intervention

Dietitians reviewed the participants’ food intake and nutritional assessments and conducted 6 biweekly telehealth or in-person counseling sessions with them. During these visits, the dietitian established individualized dietary plans that included a calorie goal, discussed challenges to dietary intake, and made recommendations for improving nutrition. If a participant did not record food intake for 5 days or more, a study coordinator contacted the participant to discuss barriers to using the Fitbit tracker and to encourage continued tracking. Study assessments were completed on REDCap (Research Electronic Data Capture; Vanderbilt University), a web-based software platform [25,26], on a paper survey, or in person using a tablet during clinic visits.

### Measures

#### Sociodemographic Characteristics

Participants’ sociodemographic characteristics were obtained from the EHR and the baseline survey. Information collected included age, sex at birth, race or ethnicity, marital status, primary language preference, whether the participant resided in an urban area (defined by matching the participant’s zip code using the 2010 US Department of Agriculture rural-urban commuting area codes) [27], 2022 Area Deprivation Index (ADI; an area-level measure of socioeconomically disadvantaged neighborhoods ranging from 0 to the 100th percentile nationally, with higher percentiles indicating more disadvantaged neighborhoods) [28], insurance type, highest educational attainment, and annual household income.

#### Clinical Characteristics

Clinical characteristics were obtained from the EHR and included tobacco use, BMI, Charlson Comorbidity Index, and cancer type or histology. The peritoneal cancer index was also measured, which grades the extent of PD on a scale from 0 to 39, with higher scores indicating a more extensive disease [29]. Eastern Cooperative Oncology Group performance status was measured, which captures the extent to which the disease affects a patient’s activities of daily living; the grades included in this study ranged from 0=fully active to 4=completed disabled [30]. Cytoreduction completeness score was measured, which captures the extent of the residual tumor, and was used to determine whether the patient underwent CRS-HIPEC for curative intent [31]. The patient’s nutritional status was measured by the Patient-Generated Subjective Global Assessment (PG-SGA) Short Form, with scores ranging from 0=no risk to 36=highest risk [32].

#### Implementation Outcomes

The data on feasibility, acceptability, and usability of the intervention were collected through objective intervention data or measured by a participant survey at the end of the intervention (week 12). Implementation outcomes were assessed against prespecified benchmarks consistent with benchmarks (60%‐70%) used in previously reported single-arm digital health interventions for patients with cancer (Multimedia Appendix 1) [33,34]. The feasibility benchmarks of successful implementation of the intervention within the GI clinic included recruitment rate (≥50%), percentage of participants who completed baseline study assessment (≥70%), percentage of participants who completed 4 of 5 study assessments (≥60%), participant retention at the end of the intervention (≥70%), participant retention at the end of the study period (≥60%), percentage of participants who attended at least 4 of 6 dietitian appointments (≥60%), and percentage of participants who logged food intake for 63 of 90 days (≥60%).

Acceptability, defined as the participant’s level of satisfaction with the intervention, was measured by the Acceptability of the Intervention Measure, a 4-item scale (score ranges 0‐20) [35,36]. A ≥70% response rate with a score >12 on the Acceptability of the Intervention Measure was used as the cutoff for establishing acceptability, indicating that participants on average had a positive experience with the intervention [35].

Usability was assessed in 2 ways. Usability, defined as the extent to which individuals were able to use the Fitbit tracker and application to log food intake, was measured by the 10-item System Usability Scale (SUS; score ranges 0‐100) [37]. A ≥65% response rate with a score >68 on the SUS was used as the cutoff, indicating that participants on average perceived the Fitbit tracker and application to be easy to use [37]. Usability of the clinical dietitian services, including the dietitian’s interpersonal skills and patient-perceived health benefits of the dietitian service, was measured by a validated 8-item scale (score ranges 0‐24) that has been used in outpatient MNT interventions for patients with cancer [38,39]. A ≥70% response rate with a score >12 was used as the cutoff for establishing acceptability, indicating that participants on average had a positive experience with the dietitian services [39].

#### Patient Outcomes

To evaluate the secondary aim of this study, patient outcomes were obtained from their EHR or study assessments and included malnutrition risk measured by the PG-SGA, health-related quality of life measured by the Functional Assessment of Cancer Therapy—General [40] and the Functional Assessment of Anorexia/Cachexia Treatment—Anorexia/Cachexia Scale [41], BMI, weight, and 1-month weight change.

### Analyses

Descriptive statistics were computed to describe the study sample and assess whether prespecified benchmarks for feasibility, acceptability, and usability were met at the end of the intervention. Given the small sample size, continuous variables were summarized using median and IQR, and categorical variables were summarized using frequency and percentage. Changes in patient outcomes at baseline and the end of the study period (week 16) were assessed using linear regressions for continuous outcomes and ordered logistic regressions for ordinal outcomes. Models included participant fixed effects to obtain within-participant estimates, and SEs were robust and clustered by the participant. We adhered to the CONSORT (Consolidated Standards of Reporting Trials) guidelines for pilot and feasibility studies and study reporting (Checklist 1) [42]. All analyses were performed in Stata (version 18; StataCorp).

### Ethical Considerations

The trial (ClinicalTrials.gov NCT05649969) was conducted at a single site, Moffitt, a National Cancer Institute (NCI)–designated comprehensive cancer center. The study was approved by Moffitt’s Institutional Review Board of Record, Advarra (protocol Pro00066098). Informed consent was obtained from all participants. To protect participants’ confidentiality, deidentified information and pseudonym IDs (eg, Participant 1) were entered into participants’ Fitbit profiles. The study staff maintained a separate password-protected file behind Moffitt’s firewall linking participant IDs to patient identifiers (eg, name and medical record number). Paper questionnaires were stored in a locked file cabinet in an office with a locked door. Only the study team had access to participant research data, and only trained staff with appropriate approvals had access to patient medical records. Participants were compensated with a US $25 gift card for completing each of the 5 study assessments; participants who completed all 5 assessments received an additional US $25 gift card.

## Results

### Sample Characteristics

**Figure 1.**
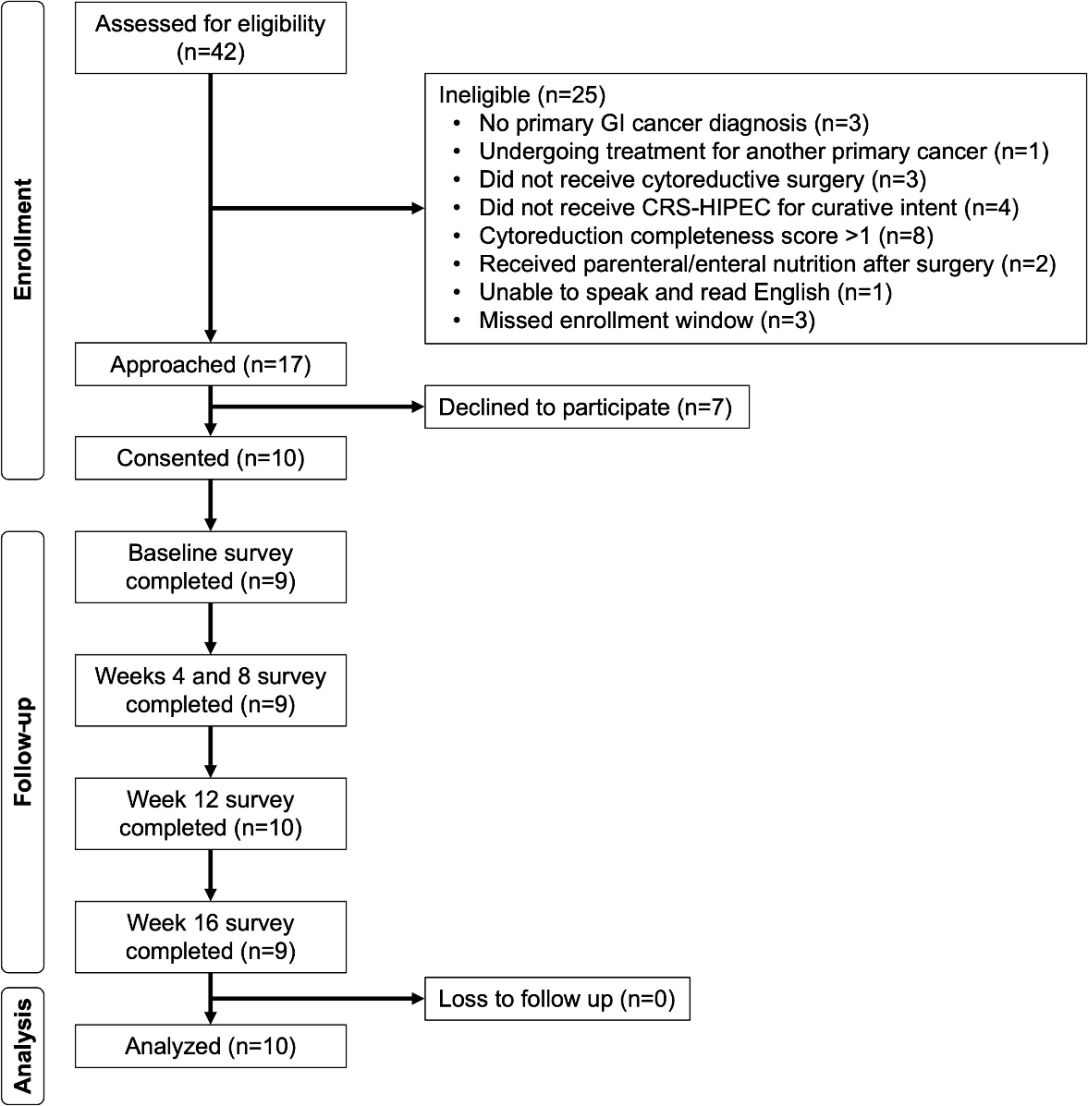
CONSORT diagram of the single-arm feasibility trial of the STRONG intervention. CONSORT: Consolidated Standards of Reporting Trials; CRS-HIPEC: cytoreductive surgery combined with hyperthermic intraperitoneal chemotherapy; GI: gastrointestinal; STRONG: Support Through Remote Observation and Nutrition Guidance.

Among the patients (n=42) screened for eligibility, 25 patients were deemed ineligible (Figure 1). Of the 17 patients approached, 10 patients consented to participate in the study. All 10 participants completed at least 1 assessment, and no participants were lost to follow-up.

Study participants had a median age of 57.5 (IQR 54.0‐69.0) years; 8 (80%) participants were female, and 10 (100%) were non-Hispanic White (Table 1). In total, 6 (60%) participants were married, 1 (10%) had never been married, and 3 (30%) were divorced. All participants spoke English as their primary language and resided in an urban area. The median ADI of participants was in the 30th percentile nationally (IQR 24-32), with a higher ADI indicating a more disadvantaged neighborhood. In total, 4 (40%) participants received health insurance through employment, 4 (40%) received public insurance, 1 (10%) had direct-purchase insurance, and 1 (10%) did not provide insurance-type information. The highest education attained by participants was some college for 3 (30%) participants, a bachelor’s degree for 3 (30%) participants, or a graduate or professional degree for 4 (40%) participants. Participants’ annual household income ranged from US $35,000 to ≥US $100,000.

In total, 8 (80%) participants never smoked tobacco, and 2 (20%) were former smokers (Table 1). The median BMI of participants was 28 (IQR 27.3-29.5), and participants had a median Charlson Comorbidity Index of 7 (IQR 7-9). A total of 6 (60%) participants were diagnosed with appendiceal cancer, 2 (20%) with colorectal cancer, and 2 (20%) with peritoneal mesothelioma. The median peritoneal cancer index was 21.5 (IQR 13.0-25.5). Participants were all fully active or able to carry out light or sedentary work, with a median Eastern Cooperative Oncology Group performance status of 0 (IQR 0-0). No participants at baseline were assessed to be at low malnutrition risk, 2 (20%) were at medium risk, and 7 (70%) were at high risk.

**Table 1.** Participant characteristics at baseline of the STRONG^a^ intervention.

Characteristic	Participant (N=10)
Sociodemographic characteristics	
Age (years), median (IQR)	57.5 (54.0-69.0)
Sex at birth, n (%)	
Male	2 (20)
Female	8 (80)
Race or ethnicity, n (%)	
Non-Hispanic White	10 (100)
Marital status, n (%)	
Never married	1 (10)
Now married	6 (60)
Divorced	3 (30)
Primary language was English, n (%)	10 (100)
Resided in an urban area, n (%)	10 (100)
Area Deprivation Index national percentile, median (IQR)	30 (24-32)
Insurance type, n (%)	
Employment-based	4 (40)
Direct purchase	1 (10)
Public (eg, Medicare, Tricare, and Veterans Affairs)	4 (40)
Unknown	1 (10)
Highest educational attainment, n (%)	
Some college, vocational training, or associate degree	3 (30)
Bachelor’s degree	3 (30)
Graduate or professional degree	4 (40)
Income (US $), n (%)	
<$35,000	0 (0)
$35,000-$49,999	1 (10)
$50,000-$74,999	1 (10)
$75,000-$99,999	2 (20)
≥$100,000	3 (30)
Unknown	3 (30)
Clinical characteristics	
Tobacco use, n (%)	
Never smoker	8 (80)
Former smoker	2 (20)
BMI (kg/m^2^), median (IQR)	28 (27.3‐29.5)
Charlson Comorbidity Index, median (IQR)	7 (7-9)
Cancer type or histology, n (%)	
Appendiceal mucinous neoplasm	6 (60)
Colorectal adenocarcinoma	2 (20)
Peritoneal mesothelioma	2 (20)
Peritoneal cancer index, median (IQR)	21.5 (13-25.5)
ECOG^b^ performance status, median (IQR)	0 (0‐0)
Cytoreduction completeness score, median (IQR)	1 (0‐1)
Patient-Generated Subjective Global Assessment Short Form score	
Low malnutrition risk (score 0‐3), n (%)	0 (0)
Medium malnutrition risk (score 4‐8), n (%)	2 (20)
High malnutrition risk (score≥9), n (%)	7 (70)
Unknown, n (%)	1 (10)
Median (IQR)	12 (9‐15)

a STRONG: Support Through Remote Observation and Nutrition Guidance.

b ECOG: Eastern Cooperative Oncology Group.

### Feasibility

Among the eligible patients (n=17) who were approached to participate in the study, 10 (59%) consented to participate (Table 2). In total, 9 (90%) participants completed the baseline assessment and completed 4 of 5 assessments. All participants completed the assessment at the end of the intervention (week 12), and 9 (90%) participants completed the follow-up assessment at week 16. A total of 7 (70%) participants attended at least 4 of 6 dietitian appointments, and 6 (60%) participants logged food intake for at least 63 of the 90 days (median logged 76, IQR 15‐87 days). Adherence to logging food intake decreased slightly over the span of the intervention, from 7 (70%) participants meeting the benchmark number of days logging food intake in the first 30 days (median logged 29, IQR 15‐30 days) to 5 (50%) participants meeting the benchmark in the last 30 days (median logged 15, IQR 0‐27 days; not shown in table).

**Table 2.** Feasibility outcomes of the STRONG^a^ intervention.

Outcome	Benchmark (%)	STRONG intervention, n/N (%)
Recruitment		
Eligible patients who consented	≥50	10/17 (59)
Study assessment completion		
Participants who completed baseline assessment	≥70	9/10 (90)
Participants who completed 4 of 5 study assessments	≥60	9/10 (90)
Retention		
Participants retained at the end of the intervention (week 12)	≥70	10/10 (100)
Participants retained at the end of the study period (week 16)	≥60	10/10 (100)
Intervention adherence		
Participants who attended at least 4 of 6 dietitian appointments	≥60	7/10 (70)
Participants who logged food intake for 63 of 90 days	≥60	6/10 (60)

a STRONG: Support Through Remote Observation and Nutrition Guidance.

### Acceptability and Usability

Among the 10 participants who completed the week 12 assessment, 8 (80%) rated the intervention as acceptable (benchmark score >12), with a median score of 18 (IQR 16‐20; Table 3). In total, 5 (50%) participants rated the Fitbit tracker and application as usable for logging food intake (benchmark score >68), with a median score of 68.8 (IQR 54.4‐90). All participants (100%) were satisfied with the dietitian services (benchmark score >12), with a median score of 23.5 (IQR 17.8‐24.0). One participant reflected on the high acceptability of the dietitian services and said, “The program added a lot of value. It helped with my recovery, especially with getting the right nutrition. It has been really great!” Another participant conveyed the value of the digital nature of the STRONG intervention, expressing “The ZOOM meetings were wonderful because I am over an hour from the hospital. The [nutritionist] was also a great encourager and contributed to my healing process.”

**Table 3.** Acceptability and usability outcomes of the STRONG^a^ intervention (N=10).

Outcome	Benchmark	STRONG intervention
Acceptability		
Acceptability of the Intervention Measure	≥70% response rate with score >12	8 (80)
Usability		
Fitbit	≥65% response rate with score >68	5 (50)
Dietitian services	≥70% response rate with score >12	10 (100)

a STRONG: Support Through Remote Observation and Nutrition Guidance.

### Patient Outcomes

Compared to baseline, average PG-SGA malnutrition scores saw a decrease of 6 points (*P*=.01), with a corresponding reduction in patients with high malnutrition risk (*P*=.03; Table 4). Functional Assessment of Cancer Therapy—General scores increased by an average of 20.5 points (*P*=.002), and Functional Assessment of Anorexia/Cachexia Treatment—Anorexia/Cachexia Scale scores increased by an average of 7.4 points (*P*=.03), indicating an improvement in participants’ health-related quality of life. There was no change in participants’ average BMI or weight, suggesting a stabilization of weight loss. This is supported by a 5.6 percentage point increase in the average 1-month weight change (*P*=.04), in which participants saw a slight weight gain compared to the previous month at week 16.

**Table 4.** Changes in patient outcomes between baseline and the end of the intervention.

Patient outcomes (n=9)	Baseline	Week 16	*P* value^a^
Patient-Generated Subjective Global Assessment Short Form score, mean (SE)	11.7 (3.8)	5.7 (4.3)	.01
Low risk, n (%)	0 (0)	3 (33)	.03
Medium risk, n (%)	2 (22)	5 (56)	.03
High risk, n (%)	7 (78)	1 (11)	.03
Functional Assessment of Cancer Therapy—General score, mean (SE)	70.4 (15.4)	90.9 (10.5)	.002
Functional Assessment of Anorexia/Cachexia Treatment—Anorexia/Cachexia Scale score, mean (SE)	28.9 (4.3)	36.3 (7.8)	.03
BMI (kg/m^2^), mean (SE)	27.5 (5.5)	26.3 (4.2)	.09
Weight (lb), mean (SE)	172.3 (29.1)	165.3 (22.0)	.09
Weight change since 1 month ago (%), mean (SE)	−5.2 (4.8)	0.4 (3.1)	.04

a *P* values of continuous outcomes were computed from linear regression models. *P* values of ordinal outcomes were computed from ordered logistic regression models. All models included participant fixed effects. SEs were robust and clustered by the participant.

## Discussion

### Principal Findings

The goal of this trial was to evaluate the feasibility and acceptability of the STRONG intervention for individuals with GI cancer and PD undergoing CRS-HIPEC. Our findings demonstrated that the STRONG intervention was feasible to be implemented with high participant recruitment, adherence, and retention to the intervention. Participants rated the intervention favorably and found the dietitian services to be both acceptable and usable. This rating is consistent with previous pilot studies assessing the implementation of mobile phone–based nutritional intervention for individuals with cancer [43,44]. Patient outcomes, including malnutrition risk, health-related quality of life, and 1-month weight change, saw marked improvement. Given that this study was a single-arm intervention without a comparison group, we were unable to attribute the changes in patient outcomes to the intervention alone without considering the effects of cancer treatment and disease progression. The successful implementation of STRONG in this study, positive feedback from participants, and promising improvements in patient outcomes suggest that a future, fully powered trial with a comparison group is warranted. Our team is currently evaluating potential improvements to STRONG and assessing alternative food logging approaches in preparation for a randomized controlled trial of STRONG.

### Comparison With Prior Work

Malnutrition screening, counseling, and related interventions remain underused in cancer care [45,46]. Nutritional counseling has been shown to improve the nutritional status, quality of life, and survival for individuals with GI cancer [47,48]. Digital nutritional interventions show clear benefits over traditional MNT, including efficiency, accessibility (eg, reduced transportation barriers), and the ability to remotely monitor patients outside of a traditional clinic visit [22]. Digital tools can also help individuals maintain adherence to nutritional interventions [14]. In our study, despite undergoing a complex surgical procedure, participants were able to adhere to the dietitian visits and food logging in the postoperative period. We hypothesize that this may be facilitated by strong support from the clinic team. Members of the surgical and dietitian teams encouraged patients to participate in the intervention and periodically checked on participants to monitor their progress through the intervention. This study is innovative in that individualized and remote monitoring of dietary needs bridges the gap between the clinical need for close, in-person patient follow-up and the substantial barriers for this patient population to access nutritional support.

Prior studies have shown that digital nutritional interventions are feasible and effective for achieving weight loss among survivors of cancer [49,50]. However, there has been limited research on the use of digital health interventions for individuals with malnutrition to maintain weight or to prevent weight loss. To the best of our knowledge, this is the first digital nutritional intervention conducted among individuals receiving CRS-HIPEC, who have increased risk for malnutrition and face unique barriers to accessing and using traditional MNT [7,20,21].

One study that assessed food-intake tracking with a Fitbit device among individuals with colorectal cancer undergoing surgery found decreased acceptability of the intervention in the postoperative period due to the complexity of the Fitbit application [51]. In our study, we also found declining adherence to tracking food intake over time. The benchmark for tracking food intake was not achieved and was driven primarily by 3 participants who logged 0, 3, and 12 days over the course of the intervention. The decline in adherence to tracking food intake was driven primarily by 2 participants who logged 12 and 24 days over the first 30 days of the intervention, followed by no additional days logged over the rest of the intervention period. It was unclear why these participants were disengaged with tracking food intake, as they did not provide any qualitative feedback.

Further research is needed to investigate why the usability of Fitbit may be low (eg, differences in digital literacy) and what strategies could be used to improve usability. One potential explanation is the choice of the usability measure. Our study team used a generic SUS that was not targeted to mobile health specifically [52]. In future studies, we plan to include mobile health–specific measures of usability to see how that may affect usability ratings. Another potential explanation is that study participants did not have sufficient education on how to use the Fitbit. Since this pilot study, our team has added teach-back sessions to the Fitbit training. Additionally, our team has created a paper version of the food log as an alternative for patients who cannot manage electronic food logging even after training. A third hypothesis is that participants may only need to food log for a certain amount of time before learning enough about their dietary patterns to manage their nutrition. Further study is needed on the optimal time needed for food logging for malnutrition self-management.

Future digital health interventions should assess the eHealth literacy of participants and make efforts to address participant concerns about the potential complexity of digital interventions [53,54]. One approach may be to target participants with low eHealth literacy with additional technical support and resources to ensure equal and inclusive participation in the intervention [53]. Another approach to improve adherence to food tracking may be to adopt a tracking system designed for patients with cancer (eg, the Automated Self-Administered 24-Hour Dietary Recall developed in collaboration with the NCI) [55]. Recent developments in image processing technology can also be leveraged to reduce the burden on participants in tracking food intake [56]. For example, artificial intelligence–enabled applications such as MyFitnessPal, Fastic, and Noom enable food tracking through capturing photos of the foods via a smartphone [56]. However, the feasibility of these technologies for individuals with cancer has yet to be fully explored.

### Limitations

Our study should be considered in light of the following limitations. First, this is a single-arm feasibility study conducted at an NCI-designated comprehensive cancer center. Therefore, the study findings may not be generalizable to other settings. Second, the study sample is small and consisted only of non-Hispanic White participants, limiting generalizability to other patient populations. There is a critical need to test the intervention in a more diverse population, across a wider range of settings, and on a larger sample. Additionally, our study focused on extending nutritional support in the postoperative setting, which has been previously recommended for individuals receiving CRS-HIPEC [57,58]. Further research is needed to test nutritional interventions for this population prior to surgery, which may improve CRS-HIPEC tolerance and reduce the likelihood of nutrition-related, postoperative complications [57,58]. Finally, this study was a single-arm trial without a comparison group. Findings on changes in patient outcomes over time cannot be solely attributed to the effect of the intervention without considering the effects of cancer treatment and disease progression. A larger, fully powered randomized controlled trial is needed to rigorously evaluate the impact of STRONG on patient outcomes.

### Conclusions

Our study demonstrated that STRONG, a digital health intervention aimed at improving nutritional management for individuals with GI cancer and PD receiving CRS-HIPEC, is feasible, acceptable, and usable. Future studies are needed to establish the effectiveness of the STRONG intervention and to evaluate its implementation in more diverse patient populations and settings.

## Supplementary material

10.2196/67108 Multimedia Appendix 1 Feasibility, acceptability, and usability scale computations and benchmarks.

10.2196/67108 Checklist 1 CONSORT (Consolidated Standards of Reporting Trials)-eHEALTH checklist.

## Abbreviations

ADI: Area Deprivation Index
CONSORT: Consolidated Standards of Reporting Trials
CRS-HIPEC: cytoreductive surgery combined with hyperthermic intraperitoneal chemotherapy
EHR: electronic health record
GI: gastrointestinal
MNT: medical nutrition therapy
NCI: National Cancer Institute
PD: peritoneal disease
PG-SGA: Patient-Generated Subjective Global Assessment
REDCap: Research Electronic Data Capture
STRONG: Support Through Remote Observation and Nutrition Guidance
SUS: System Usability Scale
