# Supplementary material for: Digital Health Intervention to Reduce Malnutrition Among Individuals With Gastrointestinal Cancer Receiving Cytoreductive Surgery Combined With Hyperthermic Intraperitoneal Chemotherapy: Feasibility, Acceptability, and Usability Trial
Source: JMIR Cancer. 2025 Apr 7;11:e67108. doi: 10.2196/67108 (PMC11996150; doi:10.2196/67108)
Supplement: Multimedia Appendix 1 [file cancer-v11-e67108-s001.docx]

Implementation outcomes were assessed against pre-specified benchmarks consistent with benchmarks (60%-70%) used in previously reported single-arm digital health interventions for cancer patients.[1, 2] Feasibility outcomes of Support Through Remote Observation and Nutrition Guidance (STRONG) intervention included recruitment rate, study assessment completion rate (at baseline and 4, 8, 12, and 16 weeks after study enrollment), participant retention rate, adherence to dietitian appointments, and adherence to logging daily food intake. Pre-specified benchmarks for the feasibility of the STRONG intervention is shown in Table S1.

**Table S1.** Feasibility outcome benchmarks of the STRONG intervention.

| **Outcome** | **Benchmark** |
| --- | --- |
| **Recruitment** |  |
| % eligible patients who consented | ≥50% |
|  |  |
| **Study assessment completion** |  |
| % participants who completed baseline assessment | ≥70% |
| % participants who completed 4/5 study assessments | ≥60% |
|  |  |
| **Retention** |  |
| % participants retained at end of intervention (week 12) | ≥70% |
| % participants retained at end of study period (week 16) | ≥60% |
|  |  |
| **Intervention adherence** |  |
| % participants who attended at least 4/6 dietitian appointments | ≥60% |
| % participants who logged food intake for 63/90 days | ≥60% |

Acceptability of the STRONG intervention was measured by the Acceptability of Intervention Measure (AIM), a 4-item, 5-point Likert scale (Table S2).[3] For each participant, the score on each question was summed to generate an individual score that ranged from 0-20. A ≥70% response rate with score >12 on the AIM was used as the cutoff for establishing acceptability, indicating that participants on average had a positive experience with the intervention.[3]

**Table S2.** Acceptability of Intervention Measure questionnaire.

|  | Completely disagree | Disagree | Neither agree nor disagree | Agree | Completely agree |
| --- | --- | --- | --- | --- | --- |
| 1. The STRONG program meets my approval. | ➀ | ➁ | ➂ | ➃ | ➄ |
| 2. The STRONG program is appealing to me. | ➀ | ➁ | ➂ | ➃ | ➄ |
| 3. I like the STRONG program. | ➀ | ➁ | ➂ | ➃ | ➄ |
| 4. I welcome the STRONG program. | ➀ | ➁ | ➂ | ➃ | ➄ |

Usability was assessed in two ways. Usability of the Fitbit tracker and application was measured by the System Usability Scale (SUS), a 10-item, 5-point Likert scale (Table S3).[4, 5] For each participant, scores were computed as follows: 1) For odd number questions, subtract one from the participant response, and 2) for even number questions, the question was reverse coded by subtracting the participant response from 5.[5] The question scores were then summed and multiplied by 2.5 to generate a score that ranged from 0-100 for each participant.[4, 5] A ≥65% response rate with score >68 on the SUS was used as the cutoff, indicating that participants had an above average perception that the Fitbit tracker and application were usable.[4]

**Table S3.** System Usability Scale questionnaire to measure the usability of the Fitbit tracker and application for tracking daily food intake.

|  | Strongly disagree | Disagree | Neither agree nor disagree | Agree | Strongly agree |
| --- | --- | --- | --- | --- | --- |
| 1. I think that I would like to use the Fitbit app frequently. | ➀ | ➁ | ➂ | ➃ | ➄ |
| 2. I found the Fitbit app unnecessarily complex. | ➀ | ➁ | ➂ | ➃ | ➄ |
| 3. I thought the Fitbit app was easy to use. | ➀ | ➁ | ➂ | ➃ | ➄ |
| 4. I think that I would need the support of a technical person to be able to use the Fitbit app. | ➀ | ➁ | ➂ | ➃ | ➄ |
| 5. I found the various functions in the Fitbit app to be well integrated. | ➀ | ➁ | ➂ | ➃ | ➄ |
| 6. I thought there was too much inconsistency in the Fitbit app. | ➀ | ➁ | ➂ | ➃ | ➄ |
| 7. I would imagine that most people would learn to use the Fitbit app very quickly. | ➀ | ➁ | ➂ | ➃ | ➄ |
| 8. I found the Fitbit app very cumbersome to use. | ➀ | ➁ | ➂ | ➃ | ➄ |
| 9. I felt very confident using the Fitbit app. | ➀ | ➁ | ➂ | ➃ | ➄ |
| 10. I needed to learn a lot of things before I could get going with the Fitbit app. | ➀ | ➁ | ➂ | ➃ | ➄ |

Usability of the clinical dietitian services, including the dietitian’s interpersonal skills and patient-perceived health benefits of the dietitian service, was measured by a validated 8-item scale, 3-point Likert scale (Table S4).[6, 7] For each participant, the score on each question was summed to generate an individual score that ranged from 0-24. A ≥70% response rate with score >12 was used as the cutoff for establishing acceptability, indicating that participants on average had a positive experience with the dietitian services.[7]

**Table S4.** Usability of the clinical dietitian services questionnaire.

|  | Not Satisfied | Satisfied | Very Satisfied |
| --- | --- | --- | --- |
| 1. I felt understood by the dietitian | ➀ | ➁ | ➂ |
| 2. The dietitian was attentive to my needs | ➀ | ➁ | ➂ |
| 3. The dietitian listed carefully to what I had to say | ➀ | ➁ | ➂ |
| 4. The dietitian came up with a good plan for helping me | ➀ | ➁ | ➂ |
| 5. The care I received from the dietitian helped my body to heal | ➀ | ➁ | ➂ |
| 6. The care I received from the dietitian has improved my general health | ➀ | ➁ | ➂ |
| 7. The care I received from the dietitian improved the results of my medical treatment | ➀ | ➁ | ➂ |
| 8. The care I received form the dietitian helped me to recover faster | ➀ | ➁ | ➂ |

Pre-specified benchmarks for the acceptability and usability of the STRONG intervention is shown in Table S5.

**Table S5.** Acceptability and usability outcome benchmarks of the STRONG intervention.

| **Outcome** | **Benchmark** |
| --- | --- |
| **Acceptability** |  |
| Acceptability of Intervention Measure | ≥70% response rate with score >12 |
|  |  |
| **Usability** |  |
| Fitbit | ≥65% response rate with score >68 |
| Dietitian services | ≥70% response rate with score >12 |

**REFERENCES**

1. Gudenkauf LM, Li X, Hoogland AI, Oswald LB, Lmanirad I, Permuth JB, et al. Feasibility and acceptability of C-PRIME: A health promotion intervention for family caregivers of patients with colorectal cancer. Supportive Care in Cancer. 2024;32(3):198.

2. Müller F, van Dongen S, van Woezik R, Tibosch M, Tuinman MA, Schellekens MP, et al. A Web-Based Mindfulness-Based Cognitive Therapy for Couples Dealing With Chronic Cancer-Related Fatigue: Protocol for a Single-Arm Pilot Trial. JMIR research protocols. 2023;12(1):e48329.

3. Weiner BJ, Lewis CC, Stanick C, Powell BJ, Dorsey CN, Clary AS, et al. Psychometric assessment of three newly developed implementation outcome measures. Implementation Science. 2017;12:1-12.

4. Bangor A, Kortum P, Miller J. The system usability scale (SUS): An empirical evaluation. International Journal of Human-Computer Interaction. 2008;24(6):574-94.

5. Lewis JR, Sauro J. The factor structure of the system usability scale. Human Centered Design: First International Conference, HCD 2009, Held as Part of HCI International 2009, San Diego, CA, USA, July 19-24, 2009 Proceedings 1: Springer; 2009. p. 94-103.

6. Vivanti A, Ash S, Hulcombe J. Validation of a satisfaction survey for rural and urban outpatient dietetic services. Journal of Human Nutrition and Dietetics. 2007;20(1):41-9.

7. Isenring E, Cross G, Kellett E, Koczwara B. Preliminary results of patient satisfaction with nutrition handouts versus dietetic consultation in oncology outpatients receiving chemotherapy. Nutrition & Dietetics. 2008;65(1):10-5.
